# Supplementary material for: A gender-based review of workplace violence amongst the global health workforce—A scoping review of the literature
Source: PLOS Glob Public Health. 2024 Jul 2;4(7):e0003336. doi: 10.1371/journal.pgph.0003336 (PMC11218983; doi:10.1371/journal.pgph.0003336)
Supplement: S4 Appendix — (PDF) [file pgph.0003336.s004.pdf]

Search Strategy for GBV-WPV Among the Health Workforce

Search Name

WPV New sources Feb- 2024

Comment

Type your comment here

Save Cancel

Set	Search Statement	Annotations	Insert	Edit	Delete
1.	faculty, medical/ or faculty, nursing/ or health facility administrators/ or hospital administrators/ or medical staff/ or nurses/ or nurse administrators/ or nurse practitioners/ or nurse specialists/ or nurses, community health/ or nurses, international/ or nurses, male/ or nurses, public health/ or nursing staff/ or physicians/ or allergists/ or anesthesiologists/ or cardiologists/ or dermatologists/ or endocrinologists/ or foreign medical graduates/ or gastroenterologists/ or general practitioners/ or hospitalists/ or nephrologists/ or neurologists/ or occupational health physicians/ or oncologists/ or ophthalmologists/ or osteopathic physicians/ or otolaryngologists/ or pathologists/ or pediatricians/ or physiatrists/ or physicians, family/ or physicians, primary care/ or physicians, women/ or pulmonologists/ or radiologists/ or rheumatologists/ or surgeons/ or urologists/	☐	☐	☐	☐
2.	Health Workforce/	☐	☐	☐	☐
3.	exp Physicians/	☐	☐	☐	☐
4.	exp Nurses/	☐	☐	☐	☐
5.	exp Midwifery/	☐	☐	☐	☐
6.	exp Gender Identity/	☐	☐	☐	☐
7.	(Gender or sex or male or female or m?n or wom?n).tw,kf.	☐	☐	☐	☐
8.	6 or 7	☐	☐	☐	☐

9.	exp Workplace Violence/	☐	☐	☐	☐
10.	exp harassment, non-sexual/ or exp bullying/ or exp incivility/ or exp "cell phone use"/ or exp permissiveness/ or exp prejudice/ or exp sexual harassment/ or exp social discrimination/ or exp social marginalization/ or exp stereotyping/	☐	☐	☐	☐
11.	exp bullying/ or exp emotional abuse/ or exp human rights abuses/ or exp incivility/ or exp social segregation/	☐	☐	☐	☐
12.	((Workplace* or occupation* or healthcare or health care or health-care or health sector or job or jobs or employment) adj3 (abus* or violen* or assault* or harass* or bully* or incivility or aggression or rape or prejudice* or marginaliz* or discrimination or permissive*)).tw,kf.	☐	☐	☐	☐
13.	((Physical or verbal or psychological or sexual or emotional or lateral or vertical or racial) adj3 (abus* or violen* or assault* or harass*)).tw,kf.	☐	☐	☐	☐
14.	9 or 10 or 11 or 12 or 13	☐	☐	☐	☐
15.	gender-based violence/ or physical abuse/ or rape/	☐	☐	☐	☐
16.	(Gender-based violence adj5 (health care or healthcare or health-care or health sector)).tw,kf.	☐	☐	☐	☐
17.	(Gender-based violence adj3 workplace).tw,kf.	☐	☐	☐	☐
18.	15 or 16 or 17	☐	☐	☐	☐
19.	(and/8) and 14 [(Gender) and (violence]	☐	☐	☐	☐
20.	or/18-19 [(gender and violence) or Gender-based violence]	☐	☐	☐	☐
21.	(Health workforce or health personnel or human resource* or physician* or nurs* or midw*).tw,kf.	☐	☐	☐	☐
22.	(physician* or allergist* or anesthesiologist* or cardiologist* or dermatologist* or endocrinologists* or gastroenterologist* or general practitioner* or gynecologist* or geriatrician* or hospitalist* or nephrologist* or neurologists* or oncologist* or ophthalmologists* or osteopath* or otolaryngologist* or pathologists* or ped?atrician* or physiatrists* or pulmonologist* or radiologist* or rheumatologist* or surgeon* or urologist* or resident* or consultant or doctor*).tw,kf.	☐	☐	☐	☐
23.	1 or 2 or 3 or 4 or 5 or 21 or 22	☐	☐	☐	☐
24.	20 and 23	☐	☐	☐	☐
25.	limit 24 to (english language and yr="2000 -Current")	☐	☐	☐	☐
26.	limit 25 to yr="2023 -Current"	☐	☐	☐	☐
27.	from 26 keep 1-530	☐	☐	☐	☐
