# Supplementary material for: A gender-based review of workplace violence amongst the global health workforce—A scoping review of the literature
Source: PLOS Glob Public Health. 2024 Jul 2;4(7):e0003336. doi: 10.1371/journal.pgph.0003336 (PMC11218983; doi:10.1371/journal.pgph.0003336)
Supplement: Attachment — Submitted filename: Review PLOS One gender-based workplace violence-Jan2024.docx [file pgph.0003336.s005.docx]

Review PLOS, workplace violence in healthcare

The authors address a very important and neglected topic of healthcare and healthcare workforce research. The methods are well justified, transparent and well described; the results are comprehensive and well presented. Major benefits of this scoping review are its broad inclusive definition of gender-based workplace violence and the analysis of a large number of studies form different geographical regions across the globe and different healthcare systems and organisational settings that provides a more complex and nuanced picture of gender-based violence at the workplace. Another important benefit is an intersectional approach that highlights that women healthcare workers are overall threatened the most by violence at the workplace, but other lines of social inequality, like race/ethnicity, intersect and may exacerbate the threats. The authors are also sensitive to the limitations of binary gender approach that dominated the studies included in the review.

The paper contributes much needed new knowledge to a very important topic. I read the manuscript carefully and with great interest but do not see any issues that would need further improvement. I think the manuscript should be published as soon as possible.
