# Supplementary material for: A gender-based review of workplace violence amongst the global health workforce—A scoping review of the literature
Source: PLOS Glob Public Health. 2024 Jul 2;4(7):e0003336. doi: 10.1371/journal.pgph.0003336 (PMC11218983; doi:10.1371/journal.pgph.0003336)
Supplement: Attachment — Submitted filename: Response to Reviewers.docx [file pgph.0003336.s006.docx]

April 22, 2024

Dear Editors and Reviewers,

Thank you for considering and reviewing our manuscript titled "A Gender-Based Review of Workplace Violence Amongst the Global Health Workforce—A Scoping Review of the Literature". We sincerely thank the reviewers for their helpful suggestions for improving our manuscript. We have resubmitted the manuscript with changes noted using "Yellow highlights" to address the reviewers' comments. We have also included our responses to the reviewers' comments below highlighted in Green. In addition, comprehensively catering to the multifaceted topic of gender-based WPV and capturing current findings, we have reviewed the search strategy (an additional 36 studies included). Most of the findings integrated were similar to those of the earlier studies. We have added a few essential aspects to the narrative results, so we have highlighted those additions in Blue for reviewers and editors. In addition, the tables and figures have been updated based on the search—revised figures have been uploaded as supplementary files and tabular information is integrated into tables in the manuscript. Similarly, the reference list is updated, and all the references/ sources are publicly available.

Thank You.

Best Regards,

**Reviewers’ Comments and Authors’ Responses**

**REVIEWER 1:** The authors address a very important and neglected topic of healthcare and healthcare workforce research. The methods are well justified, transparent and well described; the results are comprehensive and well presented. Major benefits of this scoping review are its broad inclusive definition of gender-based workplace violence and the analysis of a large number of studies form different geographical regions across the globe and different healthcare systems and organisational settings that provides a more complex and nuanced picture of gender-based violence at the workplace. Another important benefit is an intersectional approach that highlights that women healthcare workers are overall threatened the most by violence at the workplace, but other lines of social inequality, like race/ethnicity, intersect and may exacerbate the threats. The authors are also sensitive to the limitations of binary gender approach that dominated the studies included in the review.

The paper contributes much needed new knowledge to a very important topic. I read the manuscript carefully and with great interest but do not see any issues that would need further improvement. I think the manuscript should be published as soon as possible.

**Authors’ response:** Thank you for the kind feedback.

**REVIEWER 2:** The scoping review aims to report on the prevalence and risk factors of gender-based workplace violence in healthcare settings. This review provides a comprehensive picture about a very important issue but always being neglected. The searching strategies were described in detail. A total of 190 articles were included for analysis. The summarized results are presented clearly with a logical flow. The authors have done an excellent job on providing recommended strategies at individual, organizational and system levels for addressing gender-based workplace violence. Well done! I would recommend this scoping review for publication after addressing the following minor comments:

**Authors’ response:** Thank you for the kind feedback.

Page 4 line 87-88:- please report the prevalence of both nurses and physicians in Barbados (Ref13)

**Authors’ response :** Upon reviewing this study, we found that the individual prevalence of verbal abuse in these two occupational groups was not reported. However, we have add the Odds ratio (OR=2, 95% CI 1-5) reported in the paper to the our manuscript.

Line 98-101:- gender-based violence consists of both men and women. However, the definition here is for women only which may cause discrimination for men. Suggest using another definition which uses ‘individuals / persons’ rather than ‘women’

**Authors’ response:** We acknowledge that gender-based violence impacts people of all genders, including men, and we have added this in the manuscript. Still, GBV predominately affects women and we decided to keep the UN-CEDAW definition of gender-based violence because it is widely accepted. Additionally, please see lines 116-120 for our rationale for using this definition.

P.5 Line 106-109, Influence of gender on workplace violence. Apart from the references included in this section, the recent Lancet commission report – women, power and cancer also highlight the impact of gendered power relations that disproportion impact women. Suggest adding this most recent report as a reference to strengthen the argument here.

Reference:

Ophira Ginsburg, Verna Vanderpuye, Ann Marie Beddoe…Isabelle Soerjomataram, Women, power, and cancer: a Lancet Commission, The Lancet, 2023, <https://doi.org/10.1016/S0140-6736(23)01701-4>. ISSN 0140-6736.

**Authors’ response:** Thank you for drawing our attention to this important Commission. It is indeed a good example of how gendered power relations can impact women’s health. However, since it is not directly related to workplace violence, we decided not to include this reference in our manuscript.

P.9 line 218: change ‘countries’ to ‘countries/special regions’ as Taiwan is not a country. Change ‘Palestine’ to ‘the State of Palestine’ which is the official name more commonly used.

**Authors’ response:** Thank you. We have changed ‘countries’ to ‘countries/special regions’ as suggested. We did not change ‘Palestine’ to ‘the State of Palestine’ because the two studies included in this review used it as ‘Palestine’.

**REVIEWER 3:** This article is an important contribution to gathering global evidence on gender-based workplace violence. It includes a broad range of sources and presents a comprehensive overview of reports of WPV in different settings. It is timely that the gender aspects of WPV are being given greater attention in such a thorough way. The article will be useful to researchers, health care providers, civil society organisations and advocates.

**Authors’ response:** Thank you for the kind comments.

The authors may want to consider:

- Reframing the recommendation at lines 618-621, relating to strengthening legal systems' response to sexual abuse, should be broader. Suggest reviewing the recommendations on reform/ strengthening of legal systems of the "Framework Guidelines for Addressing Workplace Violence in the Health Sector", eg in section 2.1 relating to Government. There are a range of recommendations in this framework which remain highly relevant to understanding and addressing GB-WPV.

**Authors’ response:** Thank you. We have added the recommendations and additional information based on the revised search.

- Reviewing the conclusion to ensure it has not overly simplified the results of the report. For example, consider rephrasing the sentence, "Women comprise most of the health workforce, are clustered in frontline careroles and tend to be lower in the professional and organizational hierarchy. As a result of these factors, female healthcare providers are exposed to a higher prevalence of GB- WPV." This seems to undercut the idea that women face greater discrimination and violence fundamentally due to their gender - earlier in the article, gendered power relations and instrinsically patriarchal structures are more clearly acknowledged.

- Consider rephrasing the sentence, "Without attention to the gendered basis of WPV, millions of women in healthcare are at increased risk of poor health outcomes and their careers are potentially cut short due to WPV." The authors may want to reference the personal and psychological harms and breaches of human rights which women in healthcare may experience due to WPV.

**Authors’ response:** Thank you. We agreed, and we revised the conclusion to take a structural approach and addressed the recommendations.

- Consider making recommendations for further research / data required to better understand other intersectionalities which are relevant for WPV - eg sexuality, race, etc, noting that these intersectionailities are acknowledged earlier in the report.

**Authors’ response:** Thank you. We have addressed it.

**REVIEWER 4:** Reviewer #4: I congratulate the authors on their work. This was an immense effort given the limitations in the literature available on this topic, but the authors were able to draw sound conclusions and provide concrete interventions on multiple levels to address GB-WPV. My only comment is that there are many parts of the world where GB-WPV occurs but research capacity is very limited, and therefore having a formal study on it that would be published in a scientific journal (and therefore be in the databases included in this study), would be impossible. Incidents of GB-WPV, including the circumstances surrounding them can be reported in internal documents of hospitals/health systems, on social media, and in the print and non-print news, and not necessarily in scientific journals. Unfortunately, there are even instances when GB-WPV is not reported at all for multiple reasons. This is an inherent limitation of undertaking a study on this topic. Therefore, the authors could perhaps include a statement that it is likely that the prevalence of GB-WPV and the gravity of the situation, is under-reported and understated, and that we may only be scratching the surface of the ground truth. This could be tied in to their recommendations that further and more rigorous studies are needed on GB-WPV.

**Authors’ response:** Thank you. We have acknowledged the limitation and explained it with support from the revised search.
